# Dengue: A Neglected Disease of Concern

**DOI:** 10.7759/cureus.18500

**Published:** 2021-10-05

**Authors:** Joy Jisamerin, Abdulmuthalif Mohamedkalifa, Archana Gaur, Jeganathan Geetha, Varatharajan Sakthivadivel

**Affiliations:** 1 Neurology, Pushpagiri Institute of Medical Sciences, Thiruvalla, IND; 2 Internal Medicine, Government Kilpauk Medical College, Chennai, IND; 3 Physiology, All India Institute of Medical Sciences, Bibinagar, Hyderabad, IND; 4 Internal Medicine, Karpaga Vinayaga Institute of Medical Sciences and Research Center, Maduranthagam, IND; 5 Internal Medicine, All India Institute of Medical Sciences, Bibinagar, Hyderabad, IND

**Keywords:** vector-borne disease, dengue, dengue with warning signs, severe dengue, clinical features

## Abstract

Background

Dengue fever, more prevalent in Asia, is a highly neglected vector-borne disease. It has a varied presentation ranging from common fever to atypical presentation as encephalitis. This study aimed to analyze the demographic and clinical profile of dengue patients admitted to a tertiary care center in Tamilnadu.

Methodology

This retrospective study was performed by collecting patient data from the medical records department for the years 2012 to 2014. A total of 150 patients with 50 patients from each year were selected. The patient’s demographic data, clinical profile, management, and outcome were noted. Patients were divided into three groups as per the World Health Organization’s 2009 classification.

Results

Most dengue cases occurred from October to December (70.7%). The number of male and female patients was almost equal (77 [51.3%] and 73 [48.7%], respectively). The middle-aged group (21-40 years) was commonly affected (54%). The mean age was 29 ± 13.20 years. Fever was the most common symptom (100%), followed by lethargy (81.3%) and myalgia (60.7%). Overall, 10% of patients had comorbidities such as diabetes, hypertension, and ischemic heart disease. Moreover, 22.7% of patients had dengue with warning signs, and severe dengue was seen in 19.3% of patients. A significant difference was noted in the total count, comorbidities, serositis, and the duration of hospitalization between the groups. No mortality was recorded in the study population.

Conclusions

Dengue is very common in the middle-aged group. Patients with severe dengue had significant leucopenia, several comorbidities, and serositis. The mortality can be reduced to <1% and even zero in severe dengue according to our study with close monitoring and supportive care.

## Introduction

Dengue is a commonly neglected vector-borne disease with alarmingly increasing incidence in recent decades. Most of the cases are underreported due to asymptomatic and mild self-limiting illnesses. Bhatt et al. estimated 390 million infections each year, of which 96 million presented with clinical manifestations [1]. Among the total number of infections, 70% of cases were reported from Asia. Furthermore, mortality rates increased from 960 to 4,032 in 2015 [2]. India suffered from its first dengue epidemic in 1963-1964 in Calcutta and the eastern coastal region. Since then, India has faced several epidemics, possibly due to the invasion of *Aedes aegypti* throughout the country, especially in rural areas due to urbanization [3].

Dengue is a mosquito-borne viral disease caused by the dengue virus (DENV) which has four serotypes (DENV-1, DENV-2, DENV-3, and DENV-4). DENV is transmitted by a female *A. aegypti* mosquito and to a lesser extent by *A. albopictus*. Dengue can present as a flu-like illness with or without warning signs or severe dengue. There is no specific treatment of dengue. Early detection and management of severe dengue reduce mortality from 20% to 1%. Infection with a single serovar type provides lifelong complete immunity to that serovar, along with transient and partial immunity to other serovars [4].

## Materials and methods

This retrospective study was performed at a tertiary care center in southern India after obtaining ethical committee approval. Primary data were collected from the medical records department. A total of 150 patients with 50 patients from each year (2012 to 2014) were selected randomly. Patients above 13 years of age with dengue serology positive by enzyme-linked immunosorbent assay (ELISA) were included in the study. Patients aged 13 years or less, pregnant females, and patients showing coinfection with other organisms were excluded from the study. The patient’s demographic data such as age, gender, and seasonal distribution of dengue fever were noted. Clinical profiles including duration of fever and hospitalization, lethargy, myalgia, headache, bleeding manifestations, vomiting, diarrhea, and organ involvement, such as acute kidney injury (AKI), encephalopathy, and myocarditis, were observed. In addition, comorbidities such as diabetes mellitus, hypertension, and ischemic heart disease were noted. Laboratory investigations including complete hemogram, kidney function test, liver function test, prothrombin time, and ultrasonogram of the abdomen were recorded. Management of dengue and treatment outcomes were also analyzed. Patients were classified into three groups as per the World Health Organization’s 2009 classification [4]. Statistical analysis was performed using SPSS version 20 (IBM Corp., Armonk, NY). Continuous data with normal distribution were expressed as mean ± standard deviation (SD); data with skewed distribution were expressed as median and interquartile ranges. Categorical data were expressed as frequency and percentage. One-way analysis of variance and chi-square test were used for comparison among the groups. P-values of <0.05 were considered significant.

## Results

In our study, 70.7% of cases occurred from October to December, followed by 18.7% of cases from July to September. An almost equal distribution of male and female patients was noted. Most patients were in the middle-aged group of 21-40 years (54%), followed by patients in the young age group of 14-20 years (30.7%), with a median age of 26 years. The demographic data of patients are listed in Table 1.

**Table 1:** Demographic data of the patients included in the study.

Parameter	Frequency and percentage (n = 150)
Seasonal distribution	
January–March	12 (8%)
April–June	13 (8.6%)
July–September	28 (18.7%)
October–December	106 (70.7%)
Sex distribution	
Male	77 (51.3%)
Female	73 (48.7%)
Age distribution	
14–20 years	46 (30.7%)
21–40 years	81 (54%)
41–60 years	19 (12.7%)
>60 years	4 (2.6%)
Mean age (mean ± SD)	29 ± 13.20 (median 26; range 64)

Most of our patients had a longer hospital stay of more than six days (78.7%). Fever was the most common presentation (100%), followed by lethargy (81.3%) and myalgia (60.7%). Other symptoms were headache (55.3%), nausea (50.7%), vomiting (45.3%), abdominal pain (39.3%), bleeding (31.3%), retro-orbital pain (18%), back pain (12%), giddiness (6%), and diarrhea (5.3%). AKI (7.1%), encephalopathy (1.3%), and myocarditis (1.3%) were seen in few patients. Overall, 22.3% of patients had dengue with warning signs, and 19.3% of patients presented with severe dengue. Capillary refilling time was prolonged in 13 patients, and the tourniquet test was positive in nine patients. Overall, 15 (10%) patients had comorbidities including diabetes, hypertension, and ischemic heart disease. Table 2 presents the clinical characteristics of the patients included in the study.

**Table 2:** Clinical characteristics of the patients included in the study. AKI: acute kidney injury; DM: diabetes mellitus; HTN: hypertension; IHD: ischemic heart disease

Parameter	Frequency and percentage (n = 150)
Duration of hospital stay <6 days	32 (21.3%)
Duration of hospital stay 6–12 days	118 (78.7%)
Fever <7 days	133 (88.7%)
Fever >7 days	17 (11.3%)
Lethargy	122 (81.3%)
Myalgia (body pain)	91 (60.7%)
Headache	83 (55.3%)
Nausea	76 (50.7%)
Vomiting	68 (45.3%)
Abdominal pain	59 (39.3%)
Bleeding	47 (31.3%)
Petechiae	30
Gum bleeding	11
Malena	2
Epistaxis	1
hematemesis	1
Hematuria	1
Menorrhagia	1
Retro-orbital pain	27 (18%)
Back pain	18 (12%)
AKI	11 (7.3%)
Giddiness/presyncope	9 (6%)
Diarrhea	8 (5.3%)
Encephalopathy	2 (1.3%)
Myocarditis	2 (1.3%)
Dengue with warning signs	34 (22.7%)
14–20 years	12
21–40 years	17
41–60 years	5
>60 years	0
Severe dengue	29 (19.3%)
14–20 years	4
21–40 years	15
41–60 years	7
>60 years	3
Capillary refilling time <2 seconds	137 (91.3%)
Capillary refilling time >2 seconds	13 (8.7%)
Tourniquet test positive	9 (6%)
Comorbidities (total)	15 (10%)
DM	8 (5.3%)
HTN	2 (1.3%)
IHD	3 (2%)
DM/HTN/IHD	2 (1.3%)

The mean hemoglobin in our study population was 10 g/dL, and the mean hematocrit was 31%. Leucopenia of <4,000/µL was seen in 4% of cases, and thrombocytopenia of <20,000 cell/mm^3^ was seen in 5.3% of cases. Aspartate transaminase (AST) and alanine transaminase (ALT) were >40 IU/L in 6% and 21% of patients, respectively. Overall, 7.3% of patients had elevated urea and creatinine levels. All patients were immunoglobulin M (IgM) antibody positive. Hepatomegaly was present in 7.3% of the patients, and 15.3% had polyserositis. Table 3 lists the laboratory parameters of the patients included in this study.

**Table 3:** Laboratory parameters of the patients included in this study. AST: aspartate transaminase; ALT: alanine transaminase, PT: prothrombin time; INR: international normalized ratio; Ig: immunoglobulin; ELISA: enzyme-linked immunosorbent assay; NS1: Nonstructural protein 1

Parameter	Frequency and percentage (n = 150)
Hemoglobin (g/dL) (mean ± SD)	10 ± 1.45
Hematocrit (%) (mean ± SD)	31 ± 4.64
Total leucocyte count (cell/mm^3^) (mean ± SD)	5,456.67 ± 1,284.36
<4, 000 cell/mm^3^	6 (4%)
Platelet count (cell/mm^3^) (mean ± SD)	88,333 ± 83,707
≤20,000 cell/mm^3^	8 (5.3%)
20,001–40,000 cell/mm^3^	9 (6%)
40,001–60,000 cell/mm^3^	25 (16.7%)
60,001–80,000 cell/mm^3^	41 (27.3%)
80,001–100,000 cell/mm^3^	39 (26%)
100,001–150,000 cell/mm^3^	17 (11.3%)
>150,000 cell/mm^3^	11 (7.3%)
AST (IU/L) (mean ± SD)	27.8 ± 12.53
≤40 IU/L	141 (94%)
41–100 IU/L	9 (6%)
ALT (IU/L) (mean ± SD)	36.5 ± 9.5
≤40 IU/L	119 (79.3%)
41–100 IU/L	31 (20.7%)
Urea (mg/dL) (mean ± SD)	33 ± 7.49
>40 mg/dL	11 (7.3%)
Creatinine (mg/dL) (mean ± SD)	1 ± 0.22
>1.5 mg/dL	11 (7.3%)
PT (INR) (mean ± SD)	1.01 ± 0.21
>1.5 INR	9 (6%)
Serology	
IgM antibody positive – ELISA	150 (100%)
NS1 antigen positive – ELISA	14 (9.3%)
IgG antibody positive – ELISA	0
Ultrasonogram abdomen	
Normal	88 (58.7%)
Hepatomegaly	11 (7.3%)
Polyserositis	23 (15.3%)

There was no specific sex predilection in dengue, dengue with warning signs, and severe dengue. Similarly, age, hemoglobin, hematocrit, platelet count, AST, ALT, urea, creatinine, INR, fever for more than seven days, capillary refilling time of >2 seconds, tourniquet test, and hepatomegaly showed no significant difference among the three groups. There was a significant difference in the total count between dengue with warning signs and the other two groups. Most patients with severe dengue had comorbidities (21%), with a significant difference between severe dengue and dengue with warning signs. Overall, 31% of severe dengue patients had serositis, with a significant difference between severe dengue and dengue with or without warning signs. The duration of hospitalization was higher in dengue with warning signs and severe dengue. Table 4 summarizes the findings of the three groups.

**Table 4:** Group-specific characteristics of patients. AST: aspartate transaminase; ALT: alanine transaminase; PT: prothrombin time, INR: international normalized ratio * Significant difference between dengue with warning signs and dengue without warning signs;  # significant difference between dengue with warning signs and severe dengue; $ significant difference between severe dengue and dengue without warning signs.

Parameter	Dengue without warning signs	Dengue with warning signs	Severe dengue	P-value
Number of patients	87	34	29	
Male	48 (55%)	13 (38 %)	16 (55%)	0.221
Female	39 (45%)	21 (62%)	13 (45%)	0.219
Age mean ± SD	30 ± 14	26 ± 9	32 ± 15.2	0.126
Hemoglobin (mean ± SD)	10 ± 1	11 ± 1	10 ± 1.58	0.133
Total count (mean ± SD)	5,369 ± 1,200	6,003 ± 1,365*	5,079 ± 1,275.14^#^	0.010
Haematocrit (mean ± SD)	31 ± 4	32 ± 4	30 ± 5.3	0.353
Platelet count [median (Q1, Q2)]	74,500 (45,500, 98,750)	77,000 (59,000, 99,250)	75,500 (60,500, 98,000)	0.801
AST (mean ± SD)	27 ± 11	29 ± 16	28 ± 11.8	0.674
ALT (mean ± SD)	37 ± 11	35 ± 4	37 ± 7.4	0.562
Urea (mean ± SD)	34 ± 9	32 ± 4	32 ± 4.5	0.358
Creatinine (mean ± SD)	1 ± 0.2	1 ± 0.1	1 ± 0.2	0.376
INR (mean ± SD)	1 ± 0.2	1 ± 0.1	1 ± 0.2	0.723
Fever >7 days	75 (86%)	31 (92%)	27 (93%)	0.521
Comorbidities	9 (10%)	0	6 (21%)^#^	0.024
Capillary refilling time >2 second	9 (10%)	1 (2.9%)	3 (10%)	0.402
Tourniquet test	7 (8%)	0	2 (6.9%)	0.240
Hepatomegaly	7 (8%)	3 (8.8%)	1 (3.4%)	0.664
Serositis	11 (13%)	3 (8.8%)	9 (31%)^# $^	0.029
Duration of hospitalization >6 days	65 (75%)	32 (94%)*	21 (72%)	0.042

## Discussion

In this study, a total of 150 patients were included, with 50 patients from 2012 to 2014 to maintain validity and accuracy. In this study, males and females were nearly equally affected (51.3% and 48.7%, respectively). Kumar et al. and Padmaprakash et al. reported a male predominance in their studies [5,6]. Some studies have reported a female predilection as well, indicating that both sexes are affected equally [7]. The mean age in our study was 29 years. The largest age group affected was 21-40 years (54%). Elderly patients were the least affected (2.6%). There was no significant difference in gender and age between dengue with or without warning signs and severe dengue. The working population aged 21-40 years was more exposed to infection because they need to go out for work. Elderly people were mostly confined to home, making their risk of exposure low. These findings were similar to the study by Kumar et al. and Padmaprakash et al. [5,6].

Dengue cases were higher (70.7%) at the end of the year during the monsoon and post-monsoon periods. Monsoon-induced weather change and rainfall favor mosquito breeding and increase the chance of infection [8]. Fever was the most common symptom in all patients in our study (100%). The majority of the patients had a fever for less than seven days (88.7%). Most previous studies have reported fever as a common symptom [5,6,8,9]. Persistent fever beyond seven days was considered to be a sign of poor prognosis in the study by Padmaprakash et al.; however, we could not find any significant difference in patient outcomes with fever beyond seven days. Lethargy or fatigue was the next common symptom (n = 112, 81.3%), which was higher than that reported in other studies [6]. Myalgia was seen in 60.7% of cases. Padmaprakash et al. showed 77.6% of myalgia cases, whereas Lee et al. reported only 36.2% of myalgia cases [6,7]. Headache was observed in 55.3% of the study population, unlike Padmaprakash et al. who reported an incidence of 67.2%. Nausea, vomiting, and abdominal pain were present in 50.7%, 45.3%, and 39.3% of patients, respectively, which were slightly higher than those reported in other studies [9]. Bleeding was present in 31.3% of the patients. Petechia was the most common bleeding manifestation followed by gum bleeding, consistent with previous studies [8]. None of our patients had life-threatening bleeding manifestations. Retro-orbital pain and back pain was present in 18% and 12% of the study population, respectively. Giddiness and diarrhea were present in a small number of patients similar to the study by Mohan et al. [9].

Dengue with warning signs, as per WHO’s 2009 classification, was present in 22.7% of the patients (n = 34). The productive age group of 21-40 years was the most affected (n = 17, 11.3%). Severe dengue in the form of shock (n = 14) and organ dysfunction (n = 15) was seen in 19.3% (n = 29) of the patients, similar to the study by Banthia et al. [10]. Organ dysfunction in the form of AKI was present in 11 patients. Encephalopathy and myocarditis were seen in two patients each. Severe dengue was most commonly seen in the 21-40-year age group (n = 15, 10%). AKI, proteinuria, and glomerulonephritis are atypical presentations of dengue fever. In a retrospective study conducted in southern India by Mehra et al., AKI was seen in 10.8%, and leptospirosis and hepatitis were coexistent infections [11]. Myocarditis is usually caused by direct viral invasion and/or inflammatory mediators-induced damage [12]. Encephalopathy is caused by various factors such as brain edema, hemorrhage, dyselectrolytemia, organ failure, and direct viral invasion by the virus [13]. Comorbidities such as diabetes, hypertension, and ischemic heart disease were present in a small number of patients (10%) in our study. Comorbidities were significantly associated with severe dengue in our study, similar to the study by Lee et al. [14].

In our study, mean hemoglobin and mean hematocrit was 10 g/dL and 31%, respectively. Thrombocytopenia of less than 100,000 cells/mm^3^ was seen in 92.7% of patients and thrombocytopenia of <20,000 cells/mm^3^ was seen in 5.3% of patients. INR was normal in the majority of patients. There was no significant difference in these parameters between dengue with or without warning signs and severe dengue. Platelet count was not correlated with bleeding manifestation, similar to other studies [6,9]. In contrast to our study, Lam et al. showed that measurement of daily platelet count and its declining trend was effective in predicting dengue shock [15]. Leucopenia (<4,000 cells/mm^3^) was seen in 4% of patients. The total leucocyte count was significantly low in dengue without warning signs and severe dengue compared to dengue with warning signs. The change in total leucocyte count predicts the progression of dengue, as reported by Khandelwal and Khandelwal [16]. No correlation was seen between leucopenia and the severity of dengue and its complications by Jayanthi and Tulasi [17]. AST and ALT were elevated in 6% and 12% of patients, respectively. However, the maximum elevation was 100 IU/L only. Bilirubin was normal in our study. None of our patients had severe hepatitis or liver failure, which is a known complication of dengue in endemic regions. In our study, 7.3% of the study population had hepatomegaly in the abdomen ultrasonogram. However, there was no significant difference in liver enzymes and hepatomegaly between dengue with or without warning signs and severe dengue. Liver enzymes and bilirubin were significantly raised in severe dengue patients in a study by Soni et al. [18]. Polyserositis in the form of ascites and pleural effusion was present in 15.3% of patients. However, none of our patients had a respiratory compromise, which suggests that ascites and pleural effusion were minimal. Fluid accumulation is a definitive sign of plasma leakage that can be used to predict severity. In our study, serositis was significantly associated with severe dengue. Moreover, an abdomen ultrasonogram is useful in diagnosing dengue infection, especially during an epidemic, with findings of gallbladder wall edema, ascites, and pleural effusion [19].

IgM antibody was positive in all patients (100%) and NS 1 antigen was positive in only 9.3% of patients. NS 1 antigen level is usually high during the early few days of the infection. IgM antibodies appear as early as three days and remain for 30 to 60 days [20]. None of our patients had IgG positivity, suggesting that all patients had primary dengue infection. Most of our patients had six to twelve days of hospitalization (78.7%). Dengue outcome is better if patients are discharged as per the WHO criteria, which require a minimum hospitalization of one week [19]. The outcome of our study was excellent as there was no mortality, despite warning signs and severe dengue which has a mortality of 20% without treatment. This was mainly due to early recognition of warning signs and active intervention and management.

Being a retrospective study, this study has its limitations due to interobserver variability. We included only 50 patients from each year instead of all patients due to logistic reasons. The source of dengue infection such as sanitation, source of water, and locality was not included in the study.

## Conclusions

This study showed the importance of dengue, a highly neglected vector-borne disease in India, which commonly affects the productive age group of 21-40 years in the monsoon and post-monsoon period. Although fever was the most common symptom, atypical presentations such as giddiness and diarrhea were also seen. Furthermore, AKI was the most common organ dysfunction in this study. Patients with severe dengue had significant leucopenia, several comorbidities, and serositis. Excellent knowledge about common clinical manifestations, atypical presentations, and complications of dengue is necessary for early diagnosis and management. As shown in our study, mortality can be reduced to <1% and even zero in severe dengue with close monitoring and supportive care.
